# Why do pregnant women participate in research? A patient participation investigation using Q‐Methodology

**DOI:** 10.1111/hex.12446

**Published:** 2016-02-26

**Authors:** Riwa Meshaka, Stephen Jeffares, Farah Sadrudin, Nicole Huisman, Ponnusamy Saravanan

**Affiliations:** ^1^ Warwick Medical SchoolCoventryUK; ^2^ Institute of Local Government StudiesUniversity of BirminghamBirminghamUK; ^3^ Endocrinology & MetabolismWarwick Medical SchoolUniversity of Warwick & George Eliot HospitalCoventryUK

**Keywords:** consent, participation, patient choice, pregnancy research, Q‐Methodology

## Abstract

**Background:**

Patient participation in study design is paramount to design studies that are acceptable to patients. Despite an increase in research involving pregnant women, relatively little is known about the motivational factors that govern their decision to be involved in a clinical trial, compared to other patient groups.

**Objective:**

To better understand the viewpoints of pregnant women who take part in clinical trials.

**Method:**

We chose to use Q‐Methodology, a method of exploring the structure of opinions surrounding a topic. We developed a set of 40 statements that encompassed the reasons why pregnant women might want to take part in research and 30 research participants from the PRiDE study (an observational trial investigating the role of micronutrients in gestational diabetes) were asked to rank them in order of agreement. The finished matrices from each participant were compared and analysed to produce capturing viewpoints.

**Results:**

About 30 women aged 19–40 involved in the PRiDE study completed the questionnaire. There were two overarching motivators that emerged: a willingness to help medical research and improve our knowledge of medical science, and having a personal connection to the disease, therefore a potential fear of being affected by it. A third, less significant viewpoint, was that of a lack of inconvenience being a motivating factor.

**Conclusion and discussion:**

Understanding what motivates pregnant women to decide to take part in a research study is valuable and helps researchers maximize their uptake and retention rates when designing a trial involving pregnant women.

## Background

Historically, women of childbearing age have been excluded from trials due to concerns over foetal safety. There has been a relative lack of therapeutic trials involving pregnant women. This had led to a deficiency in knowledge of the safety of many medicinal products in pregnancy and in children and the prescription of unlicensed medicines whereby the prescriber takes responsibility for non‐intended side‐effects. In 1993, the FDA lifted its ban of the testing of medicinal products on women^1^ and the National Institute of Health made it a legal requirement to include women in trials.^2^, ^3^ The Royal College of Physicians followed suit in 2007 and published guidelines on how to safely involve women.^4^ Due to these changes in policy, we can expect that research involving pregnant women will be increasing, and yet little is known about why this specialist group would choose to take part.

A literature review of reasons why patients agree to take part in trials revealed a large number of studies investigating cancer patients and a smaller number investigating specialist groups including cardiac failure patients, elderly patients, low‐income groups and African Americans as specialist populations. There were few investigating pregnant women, a unique group of patients whose reasons for participation will undoubtedly differ from those already studied as a healthy group who will be paying consideration to their unborn child. We summarize the findings of these studies in Table 1.

**Table 1:** A summary of studies to date

Study	Description	Findings
Mohanna *et al*.^5^	Semistructured interviews and thematic analysis of 18 women who had declined to take part in a prophylactic nifedipine trial for preterm labour (27% uptake rate) 2 years later	Declined to take part in the trial because: Protection of the foetus ‘it will never happen to me’ Presence of a placebo arm Feeling like Guinea pigs Already ‘felt ill in pregnancy’ Not enough public knowledge of the trial
Rodger *et al*.^6^	50 cross‐sectional surveys and semistructured interviews regarding a hypothetical trial of low molecular heparin in pregnancy	Important determining factors: Potential benefit to foetus (68%) Personal health (27%) Altruism (5%) Pregnant women may be willing to accept risk to themselves if foetus could benefit.
Baker *et al*.^7^	Focus groups and semistructured interviews with 17 post‐natal women who had participated in a programme of maternity care research, followed by thematic analysis	Factors involved in decision‐making Altruism and self‐protection Enhanced care Professional guidance Suitable methodology Practical inconvenience, an apparent lack of clinical equipoise and feeling disempowered demotivated women
Kenyon *et al*.^8^	20 qualitative interviews after involvement in a randomized controlled trial of antibiotics to prevent preterm labour. Analysed using constant comparison	Experiences of the recruitment process: Motivations: better outcome for baby, helping women in the future in same situation, positive social interaction with consenting health‐care professional and high quality of information given Acuity of the situation led to perception of poor judgement of risk and understanding of trial design. Background presumption of antibiotics being safe.
Nechuta *et al*.^9^	Cross‐sectional survey in nine prenatal clinics of 311 pregnant women about attitudes to data collection for epidemiological studies involving their children	Phone interview preferred to face‐to‐face interviews Reluctance to allow access to medical records and infant examinations in women with post‐secondary school education 34–48% would require no compensation for participating.
Lyerly *et al*.^10^	22 semistructured interviews of women in H1N1 vaccine trial	Motivators: Women motivated by the media: highlighted the risks of H1N1 infection in pregnancy Perceived safety advantage Early access to vaccine To improve knowledge in the area Demotivators: risk to foetus, a placebo arm, a change to plan in care
Nechuta *et al*.^11^	311 women interviewed at first prenatal care visit about attitudes to collection and storage of biological samples (blood, placenta, cord blood)	More likely to allow collection of maternal blood (72%) than cord blood (63%) or placental tissue (64%). 68% agree with storage of samples. 25–28% would not participate even if compensated, higher in Hispanic ethnicity and primiparous women
Smyth *et al*.^12^	Semistructured interviews of 16 women involved in a trial of anticonvulsants in the prevention of pre‐eclampsia	Motivators: Unpredictability of pre‐eclampsia Quality of information received Role of health‐care professionals and family Perceived personal benefit Perception of voluntariness of joining

Studies to date that have investigated why pregnant women take part in clinical trials with descriptions and finding summaries.

There were emerging themes of note that were specific to pregnant women such as the consideration of the risk to foetus,^5^, ^10^ as well as potential benefit.^6^, ^8^, ^10^, ^12^ A theme across many of the studies was a perception that being in a trial would mean superior care to those not participating.^6^, ^7^, ^10^, ^12^ Pregnancy could be the first time that women have regular contact with health‐care professionals, so it is understandable that the attitude of the professionals had a great impact on the choices the women made.^7^, ^8^, ^12^ Altruism played a part in the decision‐making process, as it does with all types of patients considering entering a clinical trial; women want to help others, particularly those in a similar situation to them.^6^, ^7^, ^8^, ^10^ There was also a convenience factor which influenced the decision‐making process; pregnant women do not have the time nor energy to take part in trials that require a great deal of commitment, the easier it is on their schedules and health the more likely they are to accept.^5^, ^6^, ^7^, ^9^

Qualitative interviewing and closed questionnaires are commonly used to investigate subject matter relating to patient choice; however, these have limitations. Interviews are time‐consuming and can cover a broad range of subject matter. The sample size must therefore be smaller, and the results are difficult to compare and apply to a wider population. In contrast, questionnaires are more practical but are binary in their responses, and they lose the qualitative reasoning that the participant would be able to share in an interview. They also require validation by expert groups, which is a time‐consuming process. Often, people have many reasons to participate in research and both of these techniques may elucidate those reasons, but will not necessarily allow the participant to add a weighting which tells the researcher what is most to least important to them. Q‐Methodology addresses these problems. It is practical and captures the individual and varied views of a reasonable sized group, yet still allows for comparison in a quantitative manner. It also allows for the direct comparison by the participant of reasons to participate in research, allowing the researcher to place emphasis on these to design studies fitting for their chosen group of participants.

We carried out a study of women involved in a clinical trial investigating the role of micronutrients in the development of gestational diabetes. To draw particular focus to what motivated the women to consent, we used Q‐Methodology,^13^ for a systematic mapping of shared viewpoints on a topic which would not arise from interviewing or standard questionnaires alone.

## Method

The PRiDE study is a multicentre observational trial funded by the Medical Research Council investigating the role of vitamin B12 in the development of diabetes in pregnancy. Our study was formally ethically approved as a substudy PRiDE. We asked 30 women enrolled in the PRiDE study at George Eliot Hospital to take part in this questionnaire during their glucose tolerance test appointment. The inclusion criteria for the PRiDE study are same as the risk factors for gestational diabetes: BMI > 30, previously given birth to a large (>4.5 kg) baby, previously had gestational diabetes, first degree relative to diabetes, and Asian/Black Caribbean/Middle Eastern ethnic origin. To participate in PRiDE, the patient must have at least one risk factor. Women were asked to take part during glucose tolerance test clinics on 18 separate days between 2 September 2013 and 15 January 2014. All 30 women enrolled in the PRiDE study who attended clinic on one of the 18 days were asked to take part during a 2‐h waiting period and all 30 accepted.

In Q‐Methodology, the participant is asked to rank items (the Q‐set) based on their viewpoint, following a condition, for example level of agreement. The finished matrix (the Q‐Sort) is correlated with all other participants, allowing a derivation of the level of agreement and disagreement between each participant. Factor analysis is used to extract intercorrelated Q‐Sorts, which represents participants who have a similar viewpoint. The analysis is used to produce an ideal Q‐Sort for each viewpoint that is then named and interpreted.

**Figure 1:**
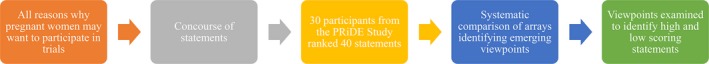
A flow chart depicting the process of collecting appropriate statements, asking participants to rank these statements and analysing the responses.

A Q‐Set of 40 statements of why pregnant women may decide to participate in the PRiDE study was derived by a literature review and informal semistructured interviewing. To capture as wide a range of statements as possible, the literature review involved all studies exploring motivational factors for involvement in research, including in non‐pregnant participants, although statements that would not apply to our population were excluded (i.e. those involving life‐saving therapy and having ‘nothing to lose’ as life expectancy was short). We identified 105 unique reasons why pregnant patients may choose to participate in trials. This was supplemented with 20 unique reasons which were derived from informal, semistructured interviewing of PRiDE researchers (including doctors, midwives, nurses and administrators) and PRiDE participants. The 125 statements were grouped into emerging themes, and 40 key statements, which best exemplified the themes, were chosen to include in the Q‐Set. This was done so that participants could work with a manageable number of items that thoroughly covered a broad range of opinions and were different enough for the participants to be able to rank. Figure 1 shows a flow chart of the methodology.

Q‐Sorts can be administered in a number of ways, including paper cards. We chose to use an iPad that was more user‐friendly, allowed the participant to carry out the ranking in stages and enabled streamlined data collection and analysis. The participants were asked to rank the 40 items using an iPad and the Poet‐Q platform.^14^ This allowed them to systematically choose the statements they agreed with most from the pool of items to form a finished Q‐Sort. The participants were then given the opportunity to explain their reasoning for the items they agreed with most and least. The questionnaire took an average of 20 min to complete. The Poet‐Q platform^15^ makes the ranking of statements user‐friendly by asking the participants firstly to group the statements into ‘agree‐most’ and ‘agree‐least’ categories, then asks the participant, in stages, to further delineate between the statements in each category. The participants were all able to complete the Q‐Sort using Poet‐Q, and the only issues encountered were with the loss of a wireless connection and with holding the iPad in the correct orientation. There was a data collector present in the department to resolve these issues. In a Q‐Sort, each statement is given a score depending on its position in the matrix. Pearson's formula is used to calculate the correlation between each finished Q‐Sort. A level of significance of *P* < 0.01 is used to flag up significant correlations in a correlation table. This was done by the PQ Method program.^16^

PQ Method highlighted emerging ‘factors’ by identifying participants who had Q‐Sorts with a high level of correlation. Each participant is compared against a ‘factor ideal’ to identify how much their ideas correlate with the standard. After the identification of the first factor, the communality they share is extracted from the matrix to find the second factor. This is done until there are no more factors left to derive. The factors were then subjected to a Varimax rotation with the intention to maximize the number of sorts showing preference for one given factor. In Q‐Methodology, the factors derived equate to statistically derived, shared viewpoints on the subject matter at hand. The terms ‘factor’ and ‘viewpoint’ are used interchangeably in the remainder of this study.

## Results

Our population included 30 women from the PRiDE study who attended their glucose tolerance test between 2 September 2013 and 15 January 2014 (Table 2). Ages ranged from 19 to 40, with 60% aged between 21 and 30. The majority (80%) of our population was Caucasian. The women had a variety of occupations, almost half in the public sector industries (Health care = 4, Community = 5, Education = 4), and more than a quarter were housewives/unemployed.

**Table 2:** Demographics

Characteristic		*N*	%total
Age	<20	1	3
	21–30	18	60
	>30	11	37
Ethnicity	White	24	80
	Mixed	1	3
	Asian	4	13
	Caribbean	1	3
Marital status	Single	5	17
	Divorced/Separated	2	7
	Married	15	50
	Common Law	6	20
	Other	2	7
Occupation	Health care	4	13
	Sales	1	3
	Education	4	13
	IT	1	3
	Community	5	17
	Arts	2	7
	Administration	3	10
	Maintenance	1	3
	Housewife	8	27
	Legal	1	3

Demographic details of the 30 pregnant women involved in PRiDE who participated in this study.

**Figure 2:**
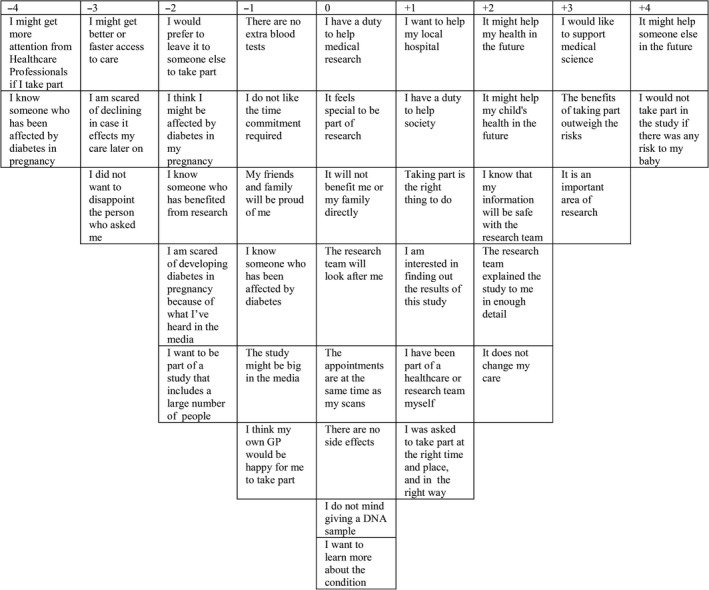
An example of a factor array (depicting viewpoint 1 in this case). A participant that loaded heavily on viewpoint 1 strongly agreed with the statements on the right and disagreed with statements on the left.

A three‐factor solution was accepted from Centroid analysis using PQ Method based on an explanation variance of 57% and eigenvalues of 14.6, 1.7 and 0.9 for factors 1, 2 and 3, respectively. A five‐, six‐ and seven‐factor solution was ruled out. A four‐factor solution was considered but ruled out as there was insufficient loading on the fourth factor. Sorts that were representative of one factor more than the others (also known as ‘loading’ on factor) with statistical significance (*P* < 0.01) were flagged as ‘factor determining sorts’. The numbers of sorts loading on each factor 1, 2 and 3 were 6, 9 and 2, respectively: a total of 17 participants were significantly loading on one of the three factors. Using the sorts that were loading on each of the three factors, PQ Method was used to create a factor array: an exemplary Q‐Sort for each factor that showed the ideal positioning of each of the statements for a participant with the viewpoint in question. An example of a factor arrays is shown in Fig. 2. PQ Method was also used to calculate the correlation between each of the factors (Table 3) and differences between them, and identified distinguishing and consensual statements.

**Table 3:** Correlations

Viewpoints	1	2	3
1	1.00	0.61	0.42
2	0.61	1.00	0.55
3	0.42	0.55	1.00

The mathematical correlation between the viewpoints calculated using Pearson's correlation coefficient. Figures are expressed in ratios with 1 delineating exact correlation and 0 delineating no correlation. We can see that viewpoints 1 and 2 are 61% correlated, viewpoints 1 and 3 are 42% correlated, and viewpoints 2 and 3 are 55% correlated.

Table 4 summaries the high‐ and low‐scoring statements for each of the derived factors. Table 5 summarizes the consensual statements between the three factors. All three types of participant were in agreement with that the area of research is an important one, and that there is trust in the health‐care professionals looking after them that their decision to take part bears no relation to the type of care they will receive. This was highlighted to them during the consenting process.

**Table 4:** High‐ and low‐scoring statements

	High‐scoring statements	Low‐scoring statements
Viewpoint 1	It might help someone else in the future	I might get more attention from health‐care professionals if I take part
	I would not take part in the study if there was any risk to my baby	I know someone who has been affected by diabetes in pregnancy
	I would like to support medical science	I did not want to disappoint the person who asked me
	It is an important area of research	I am scared of declining in case it effects my care later on
	The benefits of taking part outweigh the risks	I might get better or faster access to care
Viewpoint 2	I know someone who has been affected by diabetes in pregnancy	I might get more attention from health‐care professionals if I take part
	It might help someone else in the future	I would prefer to leave it to someone else to take part
	I know someone who has been affected by diabetes	I am scared of declining in case it effects my care later on
	I am interested in finding out the results of this study	I might get better or faster access to care
	The benefits of taking part outweigh the risks	I do not like the time commitment required
Viewpoint 3	I know someone who has been affected by diabetes	I would prefer to leave it to someone else to take part
	Taking part is the right thing to do	I am scared of developing diabetes because of what I have heard in the media
	I do not mind giving a DNA sample	The study might be big in the media
	The appointments are at the same time as my scans	I want to be part of a study that involves a large number of people
	It is an important area of research	I want to learn more about the condition

High‐ and low‐scoring statements are presented for each viewpoint. A high‐scoring statement appeared at the agree‐most end of the exemplary Q‐Sorts for each viewpoint. A low‐scoring statement appeared at the disagree‐most end. These ideas represent the defining points that make these viewpoints unique and highlight their similarities.

**Table 5:** Consensual statements

Statement	Viewpoint 1 position	Viewpoint 2 position	Viewpoint 3 position
It is an important area of research	+3	+2	+3
I am scared of declining in case it effects my care later on	−3	−3	−2
I did not want to disappoint the person that asked me	−3	−3	−2

All three types of participant agree that the area of research is an important one, that declining would not affect future care, and that they did not agree to take part to avoid disappointing the person who asked them.

### Viewpoint 1

‘Helping the future of medicine’: This viewpoint is to take part because she supports medical research the future of medicine. These participants believe that PRiDE is an important study that will help future generations. Below are example statements given by the participants.

“Cures and treatments arise when people agree to be part of medical studies so it is important that I take part to help provide answers and treatments. I am part of a healthcare team and understand how important research is to finding treatments so I feel I should help in any way I can.” Participant L5FYP2ZR is a 25‐year‐old British nurse.

“[I chose to take part] because there is no risk to me or my baby, but taking part in this study may help.” Participant SENYHCMG is a 28‐year‐old British married housewife.

These participants agree to take part for the greater good, rather than personal gain: they do not believe that it will get them more attention from health‐care professionals or that their access to care will be any better. There is also no evidence of a personal connection to the disease.

“I tend to think about the generation of the future and the impact society has on them.” Participant 7F0XALGH is a 26‐year‐old Asian youth worker.

Participants loading on viewpoint 1 were aged 25–39, 67% were British, and 50% were married. Interestingly, 67% were public sector workers, with 75% of these working in health care.

### Viewpoint 2

‘My responsibility’: in contrast to viewpoint 1, viewpoint 2 participants have a personal connection to diabetes and may feel that it is their responsibility to help the research to help those affected in the future. Comparing to viewpoint 1, these participants have a specific interest in diabetes, rather than medical research in general.

“Research is also key to my future so being part of this study may help doctors to develop a cure for diabetes sooner than hoped. Research is key to future developments in medicine and care and I am more than happy to participate in any study that will benefit my children/our future generations in years to come.*”* Participant 4ZX0Q6UE is a 34‐year‐old single administration assistant.

Due to the personal experience, there is a sense of fear amongst these participants that they may be affected.

“A friend recently had a baby and she developed diabetes during pregnancy. Before pregnancy she was fine and healthy.” Participant GAKHWRZX is a 25‐year‐old British common‐law warehouse worker.

“My auntie has severe diabetes that began at pregnancy many years ago. She is facing losing her toes this year. I used to be scared of diabetes but the amount of information now available is reassuring.” Participant E2ZJS9VF is a 31‐year‐old married British housing officer.

Similar to factor 1 participants, these participants are not interested in personal benefits of taking part in the study. Statements involving personal benefits were ranked low, as seen in factor 1.

“It doesn't matter to me if I get more care or not. I just wanted to help.” Participant CQ0YIHGD is a 29‐year‐old general assistant.

“I don't think it is right to take part in a trial to get better health care.” That's the wrong reason to do it. Participant E2ZJS9VF (as above).

Participants loading on viewpoint 2 were also 67% British and aged between 25 and 39. 78% were married (compared to 50% of viewpoint 1 participants). Once more, a significant proportion were public sector workers (56%); however, only 29% of these worked in health care.

### Viewpoint 3

‘No skin off my nose’: viewpoints in this group were more difficult to interpret about distinctive reasons that attracted them to the study; however, they did not mind taking part because they did not feel inconvenience by it. Had it been a more invasive study, they may have opted out.

“The commitment has been manageable and as my mum had diabetes I thought I might be an interesting candidate for the research. I think if one can help one should especially if the commitment is low and manageable, e.g., all extra blood tests etc. have been taken at the same time as the normal pregnancy blood tests.” Participant ZN2IRWEY is a 29‐year‐old married British housewife.

They lie somewhere between factors 1 and 2, whereby they may know someone affected by diabetes and they also think it is an important area of research, however do not seem to as feel personally responsible as factor 2 participants, or as interested in the research as factor 1 participants. They are not particularly drawn to diabetes, but their participation is not an inconvenience.

Heavy‐loading viewpoint 3 participants were aged between 29 and 34, all were British, and 50% were married. Once again, the proportion of health‐care workers was more than expected (50%).

Four statements that factor 1 participants disagreed with and factor 2 participants agreed with highlight their differing reasons for taking part as follows:

1. I am scared of developing diabetes because of what I have heard in the media
2. I think I might be affected by diabetes in my pregnancy
3. I know someone who has been affected by diabetes
4. I know someone who has been affected by diabetes in pregnancy.

It is clear that fear and personal experience have influenced the factor 2 participants.

## Discussion

We conducted a study using a methodology ideal for examining subjectivity to elucidate the opinions of women involved in an observational trial in those at risk of diabetes in pregnancy. The majority of the 30 women aged 19–40 were Caucasian, and either employed in the public sector, or were housewives. We found three distinct opinions as to why pregnant women choose to participate in research: an interest in helping medical research advancement, a personal connection to the disease and the lack of inconvenience. All three groups agreed that it was an important area of research, benefits outweighed risks, and that personal gain did not influence their decision.

As highlighted by Lylerly *et al*.,^10^ there are a group of women in whom the fear of contracting a disease motivates them to take part in research. Similarly, one group of the women interviewed by Mohanna *et al.,* 5 who had *declined* to take part in a clinical trial had done so because of a lack of belief that the disease would affect them. In our study, women who feared developing diabetes were the women who knew someone affected by the disease. It is interesting to note that these women agreed to take part, despite knowing that the PRiDE study would not help them personally. In fact, no statements relating to a benefit to personal health featured in the high‐ranking statements of any groups, unlike previous studies.^6^, ^7^, ^10^ Also in contrast to studies who noted a better outcome for the baby being a strong motivational factor^6^, ^8^ and a potential risk being a demotivator,^5^, ^10^ our study participants agreed to take part in the trial knowing that their baby would not be harmed nor helped. Interestingly, as demonstrated by Nechuta *et al*.,^11^ there was a preconception amongst the study team that cord and tissue collection and storage may be unpopular amongst pregnant women and demotivate them to take part. Our participants across all three viewpoints were indifferent about this; the collection and storage of samples did not affect their decision to participate. We postulate that including women who declined to take part in PRiDE would have brought forward this issue.

One weakness of this study is that some participants may have found the process of completing a Q‐Sort arduous, and they would have had to fill a number of other questionnaires during the same appointment. To avoid the temptation for the participants to sort statements at random, we chose to have the questionnaire administered during the 2‐h waiting period at the glucose tolerance test appointment: a time that the participants were asked to sit in a waiting room with Minimal distractions. We hoped that these measures would ensure participants paid attention to the task. The fact that there were correlations between Sorts is reassuring that the participants engaged in the process.

Another difficulty with a Q‐Methodology study is that to create a conclusive Q‐Set of statements, all current opinions on the matter need to be evaluated. We reviewed all literature to date, although this was limited; therefore, our Q‐Set may not be as broad and inclusive of all opinions as we would hope. At the end of the questionnaire, we included a free‐text section for participants to express any additional views they held. This did not reveal any further possible statements.

As evident from the demographics of the study, a large number of our participants were Caucasian, married, and either worked in the public sector or were housewives. This may represent the type of participant who would agree to take part in the PRiDE study. It is possible that housewives are able to be more flexible with their time and are therefore more open‐minded when being asked to take part in research. Public sector workers, particularly those in health care, may be more aware of the need for research in medical advancement and more eager to help. It would have been interesting to involve women who declined to take part in the study, as done by Mohanna *et al*.^5^; however, we decided against this as some women may have felt harassed if they had been asked to complete a questionnaire on involvement in research having declined to take part. It may also be true that participants who consented to take part in the Q‐Study are a group who are already more likely to want to participate in research.

In conclusion, this study has provided insight into the field of pregnant women participation in trials. The information can be used in research development for trials involving this specialist group of patients who differ from usual trial participants, because they are not unwell and they consider their unborn child when consenting to trials. We have shown that in order for a pregnant woman to consider trial participation, the study should have the potential to make a difference, be relevant to the participant and be minimally invasive in terms of time and tests. It is important to note that these women were involved in a non‐interventional trial and so their reasons may differ to those in an interventional randomized controlled trial. Further work should include investigation across socio‐economic groups and ethnicities as well as investigating the women who have declined participation to better understand their barriers.

We have shown that Q‐Methodology is a practical way to gain an objective view early on in a medical trial on what is drawing the participants to take part. It allows for fine‐tuning the recruitment process to present to potential participants the reasons that they may find attractive when making the decision to consent.

## Funding

This project was partly funded by the University of Warwick Undergraduate Research Sup‐port Scheme (URSS).

## Conflict of interest

No conflict of interest to declare.
